# Digital Twins for Monitoring Neuromotor Development in Preterm Infants: Conceptual Framework and Proof-of-concept Study

**DOI:** 10.1007/s10916-025-02252-6

**Published:** 2025-10-23

**Authors:** Sara Montagna, Rita Stagni, Giada Pierucci, Arianna Aceti, Duccio Maria Cordelli, Maria Cristina Bisi

**Affiliations:** 1 Department of Pure and Applied Sciences, University of Urbino, Urbino, Italy; 2 Department of Electrical, Electronic, and Information Engineering “Guglielmo Marconi”, University of Bologna, Cesena, Italy; 3 Department of Medical and Surgical Sciences, University of Bologna, Bologna, Italy; 4 Neonatal Intensive Care Unit, IRCCS AOU Bologna, Bologna, Italy; 5 UOC Neuropsychiatry of the Pediatric Age, IRCCS Institute of Neurological Sciences of Bologna, Bologna, Italy

**Keywords:** Preterm birth, Neurodevelopmental disorders, Digital Twin technology, Wearable sensors, Motor development

## Abstract

Preterm birth leads to an increased risk of long-term consequences, with over 50% of children born <30 weeks facing motor, cognitive, or behavioural impairments. Early monitoring of motor developmental trajectories, strongly associated with neurodevelopmental outcome, is crucial for a timely identification of deviations from the reference path and the prediction of possible neurodevelopmental disorders (NDDs). However, the current understanding of the causal pathways through which motor difficulties emerge and evolve is limited by the lack of quantitative, standardised, and interpretative measures for infant motor development, and the need for a complex multidisciplinary examination of medical history. To overcome these limitations, we propose an approach based on Digital Twins (DTs) and innovative technology-based interpretative metrics for motor assessment to support holistic longitudinal evaluations of infant development. The DT enables the integration of multimodal data, including algorithms for data processing and artificial intelligence methods for data analysis, into a unique framework. Details on the DT ecosystem, internal model, and engine are provided. As a first step, a proof-of-concept application was implemented to show the feasibility of the framework, not yet exploring its full longitudinal potential. This initial study was based on already published data (17 full-term children, 21 preterm children born between 29 and 36 gestational weeks, and 8 very preterm children born $\le$28 gestational weeks) and illustrates the integration of motor measures with clinical and cognitive information, their standardisation into the DT model, and a first set of advanced analyses. Given the relevance of the problem and the lack of standardised, structured follow-up protocols to monitor motor trajectory in preterm children, the proposed solution has the potential for a significant impact in clinical practice. Moreover, its usable and scalable design allows for easy adaptation to large, multi-center cohort studies targeting various infant clinical populations where motor function monitoring is essential (i.e. from children with rare neurological disorders to all newborns).

**Supplementary Information:**

The online version contains supplementary material available at 10.1007/s10916-025-02252-6.

## Introduction

Preterm birth leads to an increased risk of neurodevelopmental disorders (NDDs) [1], with over 50% of children born before 30 weeks facing long-term motor, cognitive, and behavioural impairments [2]. Despite advances in understanding the etiology, pathogenetic pathways, and biological underpinnings of NDDs in preterm infants, diagnosis can occur months after the first clinical signs appear, delaying prompt intervention [3]. Early detection of NDDs is one of the major challenges in child health care, with the highest potential for bringing ground-breaking changes, since the response to intervention is more significant the earlier the therapy is initiated [4]. In the first months of life, a thorough neurodevelopmental assessment combined with advanced techniques such as neuroimaging and neurophysiological tests provides important information to support the early detection of atypical development [4]. However, this level of clinical precision is only feasible in a minority of cases, typically those with significant risk factors, such as extremely preterm infants. On the other hand, clinical screening tools generally show low predictive value and reliability, particularly during the emergence and establishment of the first clinical signs [5]. This gap highlights the need for innovative solutions that enable the monitoring and early identification of NDD risk in preterm infants through widely applicable tests (e.g. for all the infants born preterm).

To this purpose, one critical area of focus is motor development, as premature exposure to the extra-uterine environment disrupts the development of musculoskeletal and nervous systems, thus potentially altering motor developmental trajectory [6]. Motor development is strongly associated with neurodevelopmental outcomes [7], as any alteration can result in mild to severe motor impairments with long-term consequences [6]. Moreover, impairments in motor, visuospatial, and sensorimotor function can contribute to cognitive delays, behavioural issues, and speech development challenges [6], given the complex interaction of social, motor, and cognitive skills (shaped by environmental factors).

Thus, early monitoring of motor development can be considered a crucial factor for predicting NDD risk in this population [2]. On the other hand, addressing the multifactorial nature of child development requires a holistic approach: early monitoring should indeed not only focus on motor development but also integrate other dimensions (e.g. cognitive, social, environmental) [8]. Moreover, only by adopting a longitudinal perspective, it is possible to capture the dynamic interplay between different factors and how they evolve and deviate over time, ensuring a more accurate identification of the risks and subject-tailored support strategies.

In this paper, we propose the adoption of Digital Twin (DT) technology as a potential solution to provide services for storing and integrating multimodal data over time, in line with the required longitudinal perspective and holistic approach. A DT is a virtual image (or replica) of some physical entity that includes models of the structure, functionalities, and behaviour of the real counterpart. As such, it is tightly linked with the physical entity by the continuous update of DT’s internal state with data acquired on the physical system, enabling it to store data on the evolution of the physical entity’s state over time. Moreover, a DT allows for collecting and integrating data of different natures, by a comprehensive and holistic data model that includes all the aspects of interest describing the physical entity [9]. The DT framework is also inherently linked to the integration of various techniques for data analytics and simulation, enabling reasoning about the current and future states of the asset while preventing undesirable outcomes [10].

When aiming to apply the DT approach for the monitoring of motor development, reliable and widely applicable measures for the longitudinal characterisation of motor performance are necessary. Current identification of potential motor impairments is based on motor-milestone history and clinical examinations. The first have demonstrated poor specificity, the latest, even when based on structured clinical assessments (e.g. General Movement Assessment, The Peabody Developmental Motor Scale-2), require trained personnel, limiting assessments only to the highest-risk children [11]. To address these limitations, quantitative approaches based on unobtrusive technology have been proposed in the literature to highlight differences between typically and atypically developing infants or children, offering a viable solution [12–14]. These methods rely on 2D/3D video capture or wearable inertial sensors (IMU) with data analysis techniques ranging from traditional human movement analysis to novel nonlinear approaches [12]. Despite the demonstrated promising results [12–19], they have remained predominantly exploratory and descriptive, as they often lack a clinical correlate with respect to the overall child development [12, 13]. In this context, their integration into a holistic and longitudinal DT framework for the monitoring of preterm development can provide a more structured approach for their clinical interpretation.

Consequently, by integrating technology-based motor assessment and advanced Artificial Intelligence (AI) methods for heterogeneous longitudinal data analysis into the unique DT framework, the contribution of this paper is a novel comprehensive approach in the context of preterm infant follow-up to monitor and understand their neuromotor trajectory and detect the risk of NDD. In particular, we present the main structure of the DT ecosystem, both in terms of internal model and of provided services. Since longitudinal data acquisition within the proposed framework is still ongoing, the proof-of-concept we are presenting in this paper relies on previously published data referring to a two-points only acquisition of multimodal information. This allows us to demonstrate how they can be standardised according to the reference Fast Healthcare Interoperability Resources (FHIR) standard^1^, and an initial set of expected analytical and predictive outcomes by integrating and processing motor, cognitive, and clinical data. Although preliminary, this first evaluation provides a first insight into the potential of the proposed approach.

## Materials & Methods

In this study, we propose a DT-based framework for the efficient management of multimodal data acquisition, preprocessing, and analysis, specifically designed in the context of premature newborns. This approach exploits the power of DT technology to create a dynamic, virtual representation of the infant, enabling more precise monitoring and risk identification. Through this framework, our goal is to integrate various data sources and longitudinal data acquisition with AI-based methods of data analysis. The long-term perspective is to enable a comprehensive understanding of the neuromotor development of preterm infants and support clinical decision making.

### A Digital Twin Ecosystem for Infants

**Fig. 1:**
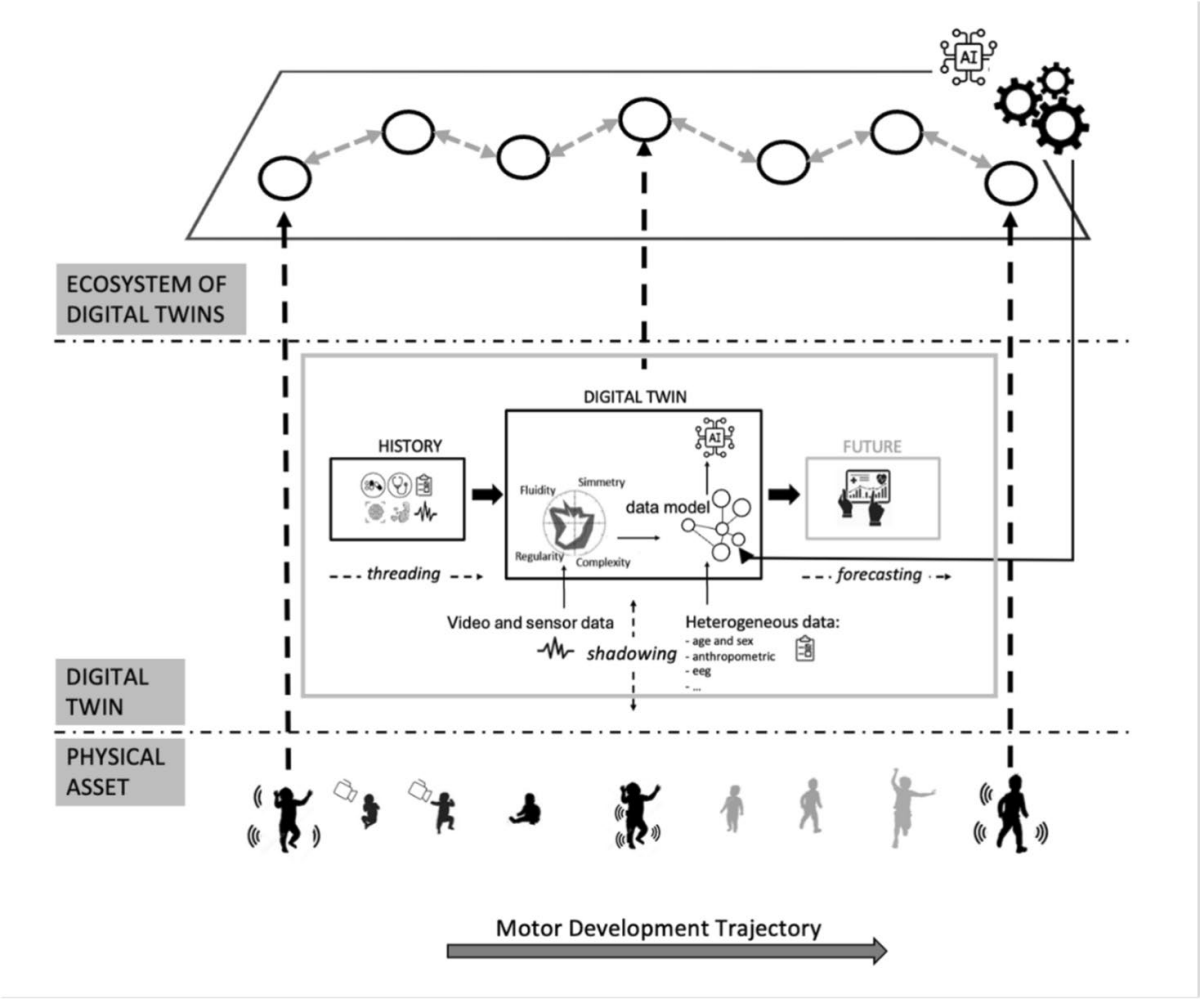
DT ecosystem of infants

In this section, we provide a description of an ecosystem of DTs devised for monitoring infants during their development. Assuming to adopt DTs as a pervasive approach, we model this scenario as an ecosystem of connected DTs, each one mapping an infant (Fig. 1). Each infant can be mirrored into its human DT [20] which represents a counterpart in cyberspace of the real person, recording each information needed, from general information (age, sex, weight) to more specific information strongly related to the objectives of this study, and reported in Tables 1 and 2. Data of each examination, motor assessment, treatment, nutrition can be transferred to the digital space and stored. This information is kept continuously updated and aligned with the state of the real system, or acquired at specific timestamps. In particular, given the sensitivity of the patients we are tracking, and the adoption of an ad-hoc instrumentation needed to acquire data, a continuous monitoring of all the variables tracked is not necessary, or even harmful. However, the longitudinal monitoring over clinically relevant timestamps is still guaranteed. The resulting network of DTs represents the state of the infant population at any given time, enabling aggregate analyses and being a source of data for training machine learning (ML) algorithms.

### The DT Internal Model

Although a similar ecosystem model can be applied in various medical contexts in paediatric population, this paper specifically focuses on preterm infants at risk of NDDs. The DT internal model is thus configured accordingly: it is designed for a general infant and includes the collection of data relevant to the specific issue but potentially available for any infant. Our primary focus is on preterm infants ($<37$ weeks gestational age), with particular attention to very preterm infants ($<32$ weeks gestational age) and very low birth weight infants ($<1500$ *g*), who face the highest risk of NDDs. These infants are evaluated from birth to 24 months corrected age (CA) —age calculated not since the actual birth but since time of expected delivery, also known as term-equivalent age (TEA). This period defines the time span considered for the DT. Each DT manages each kind of available information on infants, possibly heterogeneous, and then is dynamically fed with newly acquired data thus realising a complete history on infant state from birth to 24 months CA. Specifically, for the population of interest in this paper, we identified the following clinical data and quantitative metrics assessing motor functions that must be acquired and stored in our DTs.

#### Longitudinal Clinical Data

Longitudinal clinical data recorded at TEA, and at 3, 6-, 12-, 18- and 24-months CA (T0, T3, T6, T12, T18 and T24), are acquired and reported in Table 1 under seven domains. These data are those that are currently being collected as part of the ongoing follow-up, and the timeline is conducted in accordance with the Italian national guidelines, as outlined in the document provided by the Italian Society of Neonatology (SIN).^2^

**Table 1:** Clinical data

Category	Details
Pregnancy and delivery	Maternal comorbidities before and during pregnancy, length of gestation, mode of delivery
Early neonatal period	Apgar score, resuscitation in the delivery room, early-onset sepsis
Neonatal stay in the NICU	Length of hospital stay, major comorbidities
Brain imaging data	Brain imaging data obtained using cranial ultrasound from birth to TEA, brain magnetic resonance imaging at TEA
Growth data	During hospital stay, at discharge and during follow up
Nutritional data	During hospital stay, at discharge and during follow up
Developmental status (cognitive, language, and motor development)	Hammersmith Infant Neurological Examination at T0 and T3, Bayley Scales of Infant and Toddler Development, Third Edition (Bayley–III; Bayley, 2006), from T6 to T24

#### Longitudinal Data for Motor Assessment

Longitudinal data measuring motor functions are acquired on specific milestone for motor development, depending on their age, using novel technology-based assessment [12–14]. Table 2 describes the tasks analysed and corresponding time of assessment, explored neuromotor function, and selected instrumentation. Chosen technologies guarantee usability and acceptance in the clinical context. In particular, kinematic data are extracted from videos at T0 and T3 using open pose estimation technology (DeepLabCut [21]) and directly from IMUs from T6 to T24, then used as inputs for the DTs.

**Table 2:** Motor control data collection and instrumentation for different developmental stages

	Time of Assessment	Explored Neuro Motor Function	Task	Instrumentation	Kinematic Data Extraction
General movements	T0-T3	Central pattern generated spontaneous motor behaviour	Infant in supine position in a standard cot, recorded for 5 min	2D video camera	2D segmental kinematics through human pose estimation software
Independent sitting	T6-T12-T18-T24 (when present)	Motor control of the trunk during sitting	Infant in independent sitting position for 1 minute (or less, depending on participant’s abilities)	IMUs on the trunk, on the wrists and on the lower legs	3D trunk acceleration signals (variable of interest) and 3D upper and lower limb acceleration and angular velocity signals (to control for possible limb movements)
Independent Crawling	T6-T12-T18-T24 (when present)	Gross motor control during crawling	Infant crawling in a corridor at self-selected speed ($>10$*m* or less, depending on participant’s abilities)	IMUs on the trunk, on the wrists and on the lower legs	3D trunk acceleration signals and 3D upper and lower limb acceleration and angular velocity signals
Independent standing	T12-T18-T24 (when present)	Gross motor control of the trunk during standing	Infant in independent standing position for 1 minute (or less, depending on participant’s abilities)	IMUs on the trunk, on the wrists and on the lower legs	3D trunk acceleration signals (variable of interest) and 3D upper and lower limb acceleration and angular velocity signals (to control for possible limb movements)
Independent walking	T12-T18-T24 (when present)	Gross motor control of the trunk during walking	Infant walking in a corridor at self-selected speed ($>15$ *m* or less, depending on participant’s abilities)	IMUs on the trunk, on the wrists and on the lower legs	3D trunk acceleration signals and 3D upper and lower limb acceleration and angular velocity signals

**Fig. 2:**
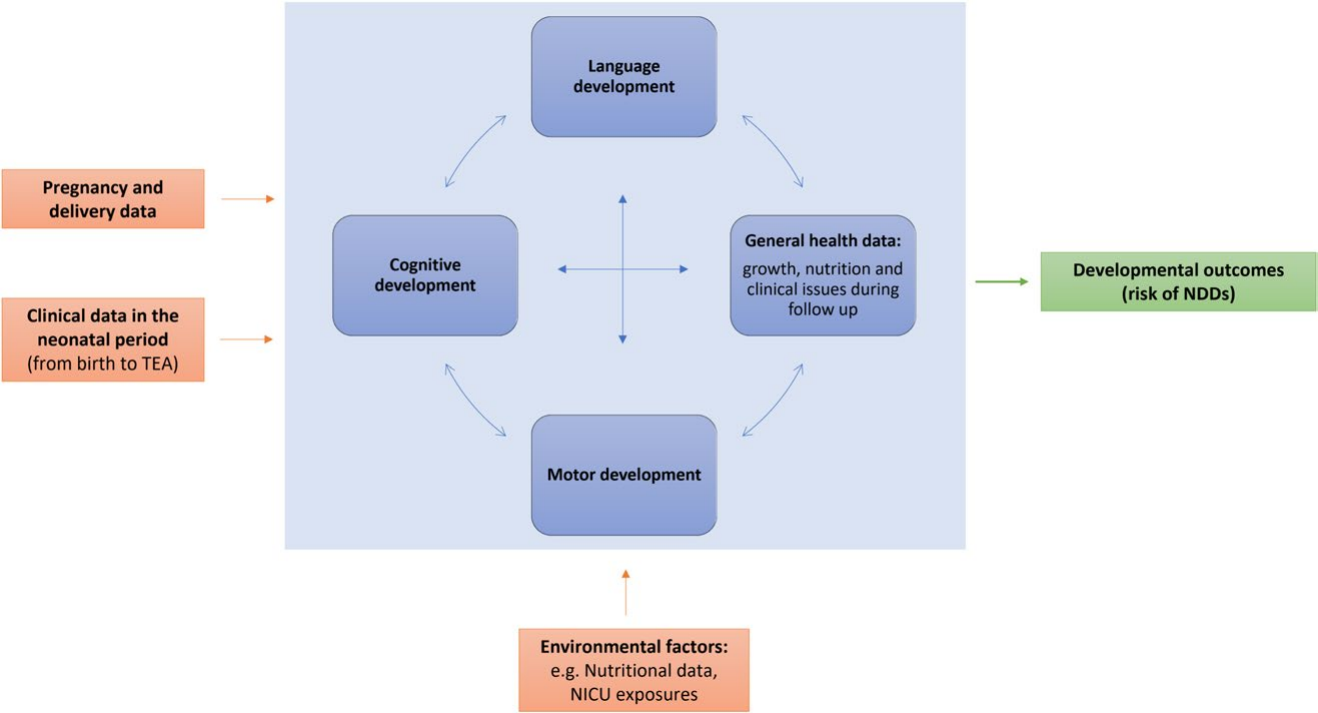
Knowledge Graph showing relation among concepts and data in the proposed DT framework

To facilitate the availability, exchange, integration and management of information, data are stored according to established protocols and standards, such as the FHIR which emerged as a reference to achieve interoperability in healthcare systems [22]. Given the diversity of collected data and the continuous evolution of the DT model, a data structure to manage these changes and express semantic relationships between concepts is needed. Domain-specific knowledge graphs are thus defined to map interactions between various pieces of information, allowing higher-level analyses [23]. They integrate personalised information with general healthcare concepts. This is achieved through semantic tools like ontologies (e.g., SNOMED-CT) and logical rules (e.g., SWRL). In Fig. 2, a first version of the knowledge graph representing the main concepts for modelling the DT of infants is presented.

### DT Internal Engine

Looking into each DT, the state of each infant can be inspected, and different kinds of analysis can be performed on the acquired data. A specific module is designed for data preprocessing, including algorithms for the quantitative assessment of infant motor competence, while the cognitive service component supports different types of analyses, from what-if to risk detection and decision making.

#### Data Processing for Quantitative Motor Assessment

Quantitative, interpretable metrics of motor performance are extracted from the kinematic data for each analysed task, obtained from videos or sensors. The following dimensions of motor performance are considered: *(i)* temporal parameters, *(ii)* temporal variability, *(iii)* motor complexity and automaticity. Based on the literature [19, 24–26], different algorithms are implemented for each dimension.

#### Data Analysis

The DT includes a multimodal analytical quantitative analysis that integrates motor and clinical data to support both clinical evaluation and the longitudinal assessment of how quantified motor competencies evolve across different tasks, characterising motor control maturation in this population. Preprocessing techniques are incorporated into each DT to address various tasks, such as missing data imputation by mean or median substitution and class imbalance correction by random undersampling or SMOTE [27]. Different ML algorithms are trained – and periodically updated and fine-tuned with newly acquired data – to enable early detection of NDD, identify risk factors and provide decision support. These algorithms include methods supporting clustering, such as k-means , and classification tasks, such as tree-based models. Methods to guarantee algorithms interpretation are integrated: they mainly extract feature importance ranking, for instance by computing SHAP values. Moreover, higher level methods are fed by the KG representation of semantic concept, with the goal to automatically infer new knowledge about the patient and/or the population, highlighting causal relationships.

To conclude, in designing and implementing the model, particular attention is paid to data-protection issues. Any data acquired and then used in the analyses are treated with heightened sensitivity, given the need to adhere to legal and ethical standards. In particular, mechanisms to pseudoanonymise data are included, and all the algorithms are developed to operate without the need for personally identifiable information.

## Proof-of-Concept

### Dataset

For a first proof-of-concept application, we exploited data collected and presented in [19]. Data refer to forty-six children: 29 born preterm ($\le 37$ weeks gestational age), and 17 full-term controls, with comparable median and range of age and walking experience and include information referring to two time points: birth and T24. These data do not include all the longitudinal and multimodal information on clinical and motor function mentioned above. However, they do contain heterogeneous data (from the domains of pregnancy and delivery, growth data, developmental status, and motor data) which allow us to demonstrate the feasibility and advantages of the proposed approach. The following information are available for each child:

*Clinical data:* sex, gestational age in weeks, twin status, growth data (length and body weight at birth and at T24), walking experience (in weeks), BSID-III cognitive standardised scores;

*Motor assessment of independent walking:* raw collected data from IMUs positioned on the trunk and on the lower legs (3D acceleration and angular velocity signals).

Complete and detailed information for each child is provided in [19]. Using FHIR resources, we were able to model the patient’s clinical and motor data. A simplified example of the FHIR RDF model for a patient is available in Listing 1. It is intended to illustrate the core concept clearly and may omit some syntactic details required by the full FHIR standard.

**Figure:**
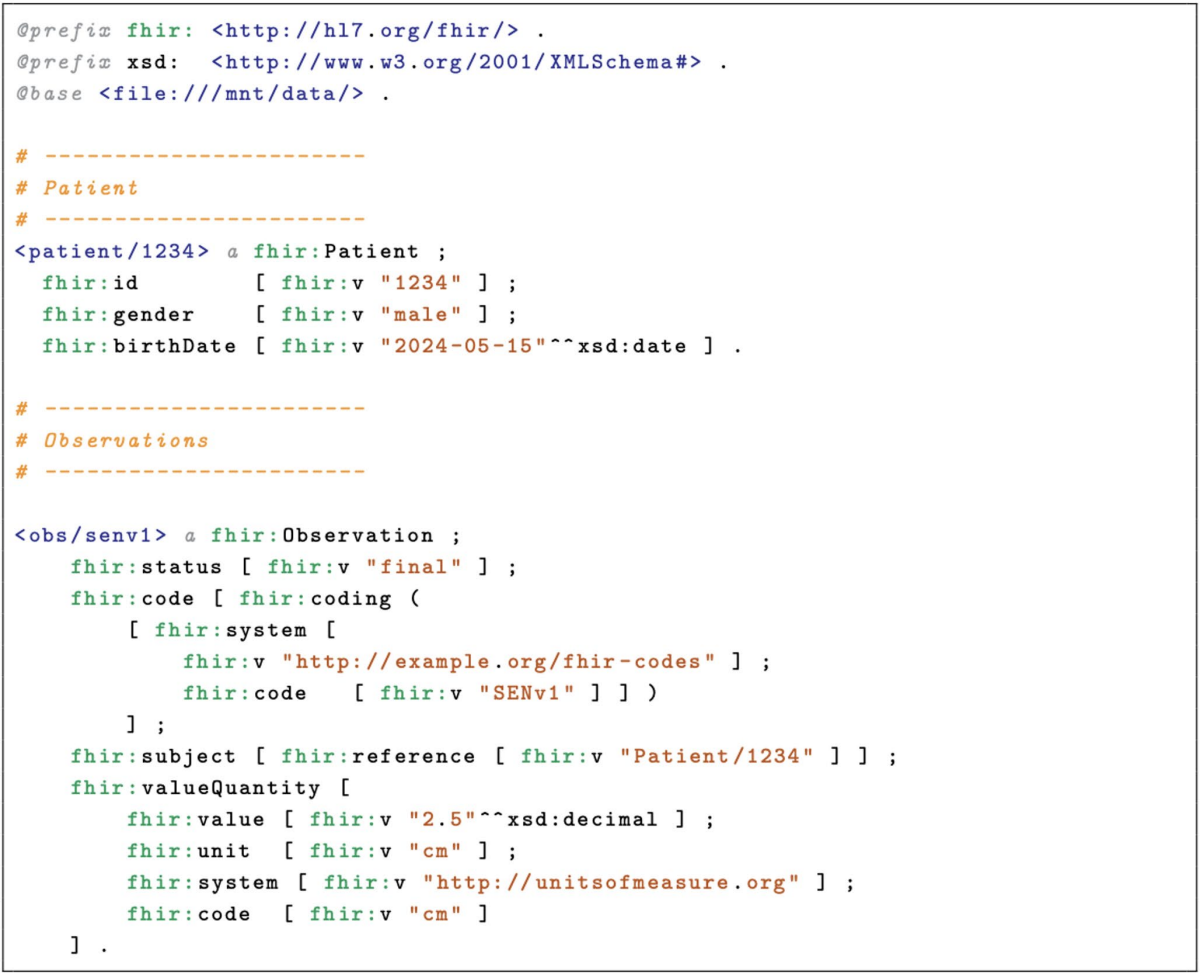

### Objectives of the Analyses

The primary objective of this proof-of-concept study was to demonstrate how data can be modelled and standardised according to the FHIR standard, how they can be automatically preprocessed and a set of ML-based analyses that can be performed with the proposed approach despite the limited sample size and the lack of longitudinal data of motor assessment. In the previous work [19] the objective was to highlight potential differences in locomotor performance between full-term and preterm toddlers, by using standard statistical analysis. In this work, by exploiting the proposed DT framework, we aimed to enhance the analysis of the available data, focusing on identifying which factors, among clinical and motor ones, best predict the highest risk of motor delays in this population. Thus, preterm children born at $\le 28$ [28] gestational weeks and/or with $\le 1000$ *g* [29] of body weight were considered at the highest risk ($n = 8$), and all the other children (preterm and full-term) at low risk ($n = 38$).

Given the limited sample size and the absence of longitudinal data of motor assessment, the primary objective of this case study was to demonstrate the automated data processing and the range of advanced analyses that can be performed with the proposed approach. However, the results will be discussed as preliminary findings.

### Methods

All the clinical data mentioned in Section Dataset were considered, except for birth weight and gestational age, as these were used to define the high-risk class.

Sensor data was processed according to [19]. The gait performance metrics [19, 24, 30], described in Table 3, were calculated for each participant. Temporal parameters, Motor Quality metrics (except for Harmonic Ratio) and Variability metrics were extracted from the angular velocities of the legs; Motor complexity and Automaticity metrics, and Harmonic Ratio were calculated on trunk acceleration components, separately (vertical V, mediolateral, ML, and antero-posterior, AP).

**Table 3:** Sensor based metrics of gait performance

Measure	Acronym
Temporal Parameters	
Stride time (s)	StrideT
Normalized stride time (adimensional)	nstrideT
Step time (s)	stepT
Normalized step time (adimensional)	nstepT
Stance time (% of StrideT)	stanceT
Double support time (% of StrideT)	DS
Motor Quality	
Harmonic Ratio (V, ML, and AP)	HR (v, ml, ap)
Stride Symmetry	symm_stride
Step Symmetry	symm_step
Variability (of temporal parameters)	
Short term variability via Poincarè plots	SD1
Long term variability via Poincarè plots	SD2
Standard deviation	std
Motor Complexity and Automaticity	
Multiscale Entropy (Sample Entropy of trunk acceleration components (V, ML, and AP) at time scales ($\tau$) from 1 to 6)	SEN (v, ml, ap) (1-6)
Recurrence Quantification Analysis of trunk acceleration components (V, ML, and AP). Extracted metrics: Recurrence Rate (RR), Determinism (DET), and Average diagonal Length (AvgL)	RR, DET, AvgL (v, ml, ap)

During the data preparation phase, normalisation exploited the Min-Max Scaler, while class imbalance in supervised algorithms has been addressed using two distinct approaches: by automatically computing class weights inversely proportional to their frequencies in the dataset, and by applying SMOTE to generate synthetic instances and augment the minority class.

For the analysis, different methods have been adopted:

- *t-SNE* This visualisation is employed to project high-dimensional data into a two-dimensional space. Each point depicted in the visualisation corresponds to a data instance, with its colour indicating the respective class membership. This visualisation method is crucial as a first verification step towards the search of latent patterns and structures within the dataset, thereby facilitating interpretation and analysis. Typically, instances with similar features tend to cluster in close proximity.
- *Clustering* It is performed using k-means with $k=2$. To ensure statistical significance, 30 iterations were carried out. Moreover, the Kernel Density Estimation (KDE) distribution of the most significant features has been performed, estimating the density, useful to provide a deeper understanding of the feature distribution within each cluster.
- *Classification* A set of supervised machine learning algorithms is applied for the classification task of identifying high vs. low-risk infants. Specifically, among linear models, we utilised Support Vector Machines and Logistic Regression. For tree-based methodologies, Decision Tree is employed, while for ensemble methods, we applied Random Forest and Extreme Gradient Boosting (XGBoost). The data set was divided into training sets (75%) and testing sets (25%). Training was performed using a Randomized Search with $k=5$, i.e. 5-fold cross-validation, to identify the optimal model, with an emphasis on balancing errors according to the class distribution. Additional experiments were conducted using SMOTE. To enhance interpretability and identify the most important features, the SHAP method was used.

### Results

#### t-SNE

**Fig. 3:**
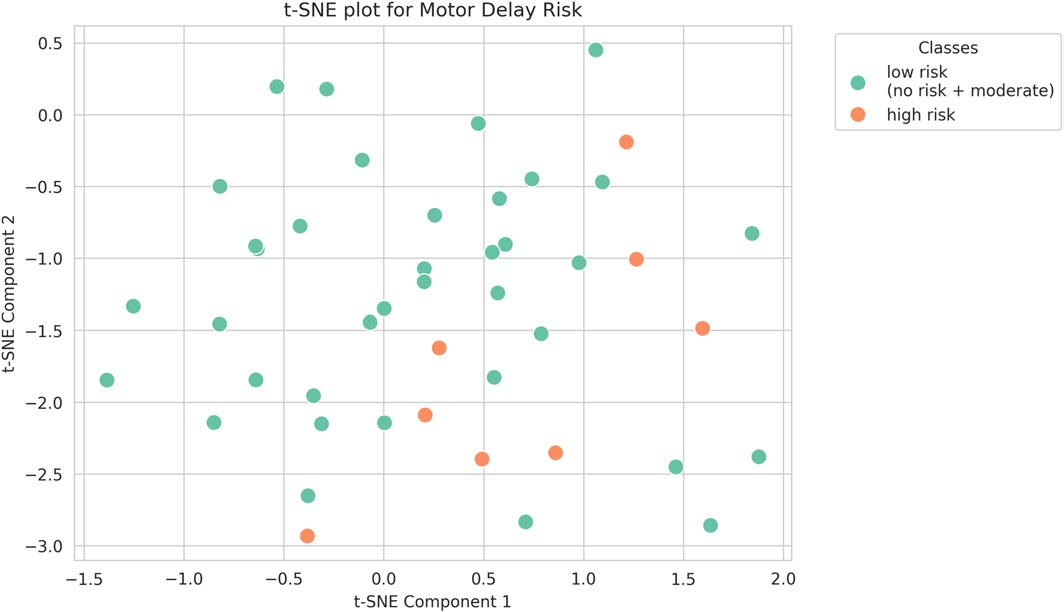
tSNE - Low vs High

Figure 3 reports the application of t-SNE to our dataset, showing inherent complexities, as evidenced by the observed overlap between the two distinct classes. However, high risk infants are distributed only in the right and lower quadrant space, revealing a potential pattern inherent in data.

#### Clustering

**Fig. 4:**
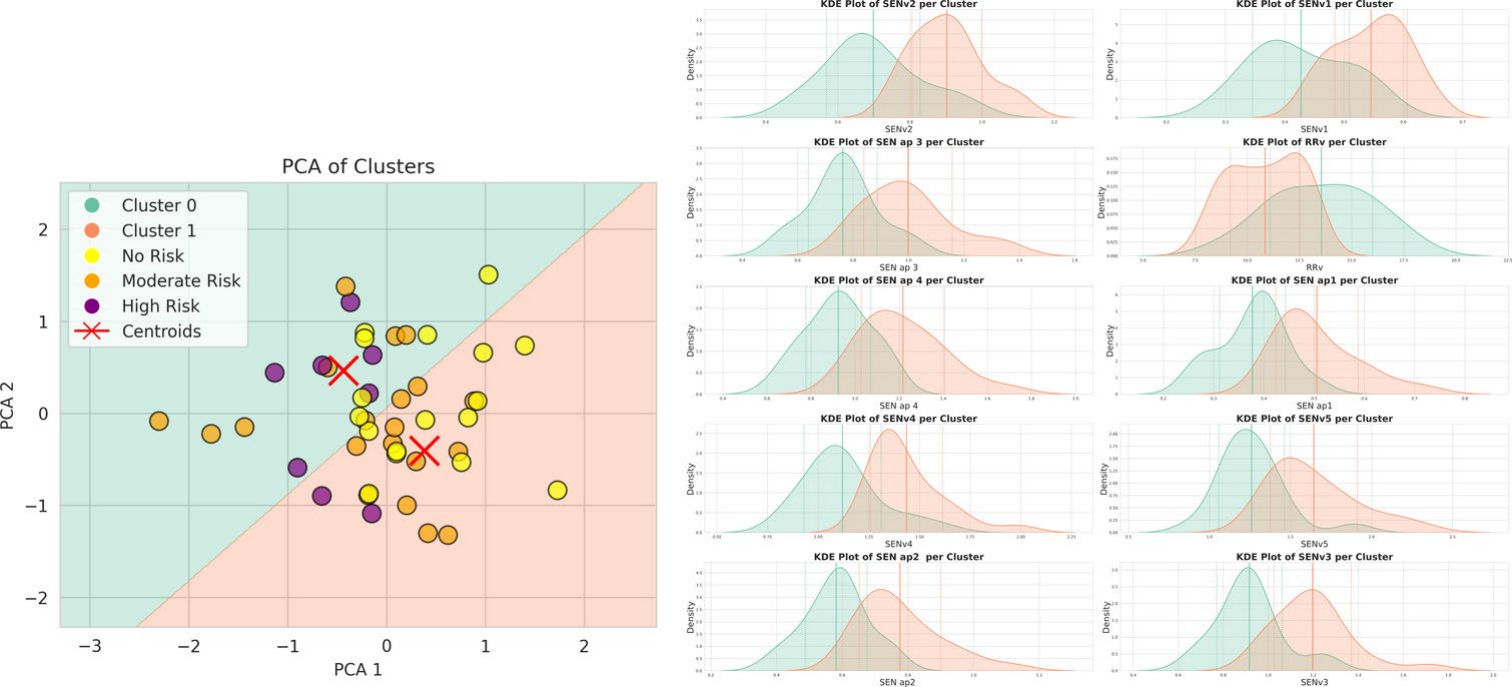
Cluster with PCA (left) and top feature KDE plot distribution by cluster (right)

**Fig. 5:**
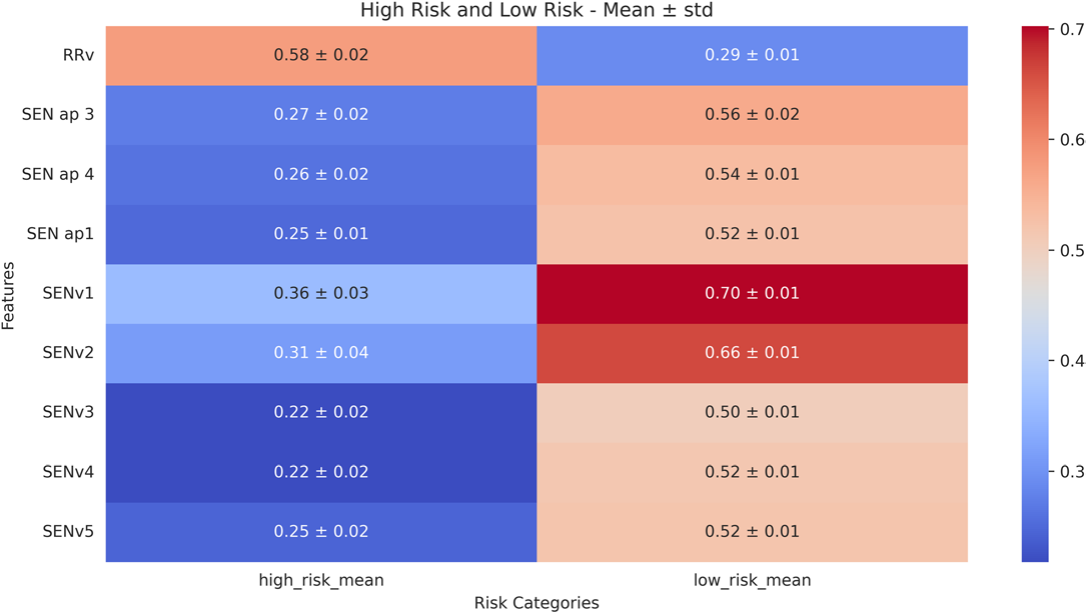
Feature Heatmap

In Fig. 4, the result of a clustering run is shown together with the KDE distribution of the most important features. For interpretation purposes, children at the highest risk based on clinical criteria (i.e., born at $\le 28$ gestational weeks and/or with a birth weight of $\le 1000$ *g*) are represented with purple circles, preterm children born between 29 and 36 gestational weeks (moderate risk) in orange, and full-term children in yellow. Moreover, Fig. 5 illustrates the mean and standard deviation of the feature importance values, averaged over the 30 runs conducted, demonstrating the robustness of the results across these iterations.

By identifying the cluster containing the highest number of children at high risk based on clinical criteria (Fig. 4 left, purple circles, $n = 6$), the DT allows to determine the features that best represent this cluster, enabling clinical discussion and interpretation of these results in relation to child development. In this cluster, 75% of children at high risk, 42% of children at moderate risk, and 35% of children at apparent no risk (full-term children) are present.

By analysing additional clinical data of preterm infants which had not been included as features in the analysis, we observed that:

- the two high-risk children (purple circles) in the low-risk cluster were born at 28 weeks of gestation, while those in the high-risk cluster between 23 and 27 weeks;
- children at moderate risk (orange circles) in the high-risk cluster had a longer hospital stay at birth (median [min-max], 54 [16-83] days) than those in the low-risk cluster (median [min-max], 30 [12-50] days).

These findings highlight the potential of including a wide set of detailed clinical variables as well as quantitative metrics in refining early identification strategies and understanding causal pathways of deviations from the typical developmental trajectory. In particular, it can be observed that nonlinear metrics of motor performance (i.e., SEN and RQA) on the sagittal plane distinguish the two clusters. Children in the high-risk cluster show an increased pattern automaticity in the vertical direction (RRv) and a decreased motor complexity (SEN) in the anteroposterior and vertical directions, suggesting a more automatic and less complex gait pattern on the sagittal plane. In agreement with literature [19], this corresponds to a simpler early form of gait and a possible delay in the development of flexibility and the ability to perform complex movements [31].

#### Classification

Table 4 reports the classification results, according to the main evaluation metrics, obtained across the five algorithms tested and using weighted error to address class imbalance. The additional experiments conducted using SMOTE for oversampling the minority class did not yield a significant performance improvement, and the results were broadly comparable to those obtained with class weighting. Among the models evaluated, XGBoost outperforms all others with the highest values across multiple metrics, including a recall equal to 1.0. This result makes it the most effective model in this comparison. Logistic Regression, Decision Tree, and Random Forest achieve similar performance. On the other hand, SVM model shows significantly poor results, with values close to zero across all metrics. These low scores can be interpreted as an indication of the model’s inability to effectively capture the underlying patterns of the data, suggesting potential issues in its configuration or the nature of the data being unsuitable for this model.

**Table 4:** Model Performance Comparison – bold indicates the best performing model

Model	Acc	BA	Precision (1)	Recall (1)	F1 Score (1)	AUC (1)	MCC
Logistic Regression	0.83	0.90	0.50	1.0	0.67	1.0	0.63
Decision Tree	0.83	0.90	0.50	1.0	0.67	0.90	0.63
Random Forest	0.75	0.65	0.33	0.50	0.40	0.90	0.26
XGBoost	**0.92**	**0.95**	**0.67**	**1.0**	**0.80**	**0.95**	**0.77**
SVM	0.83	0.50	0.00	0.00	0.00	0.00	0.00

**Fig. 6:**
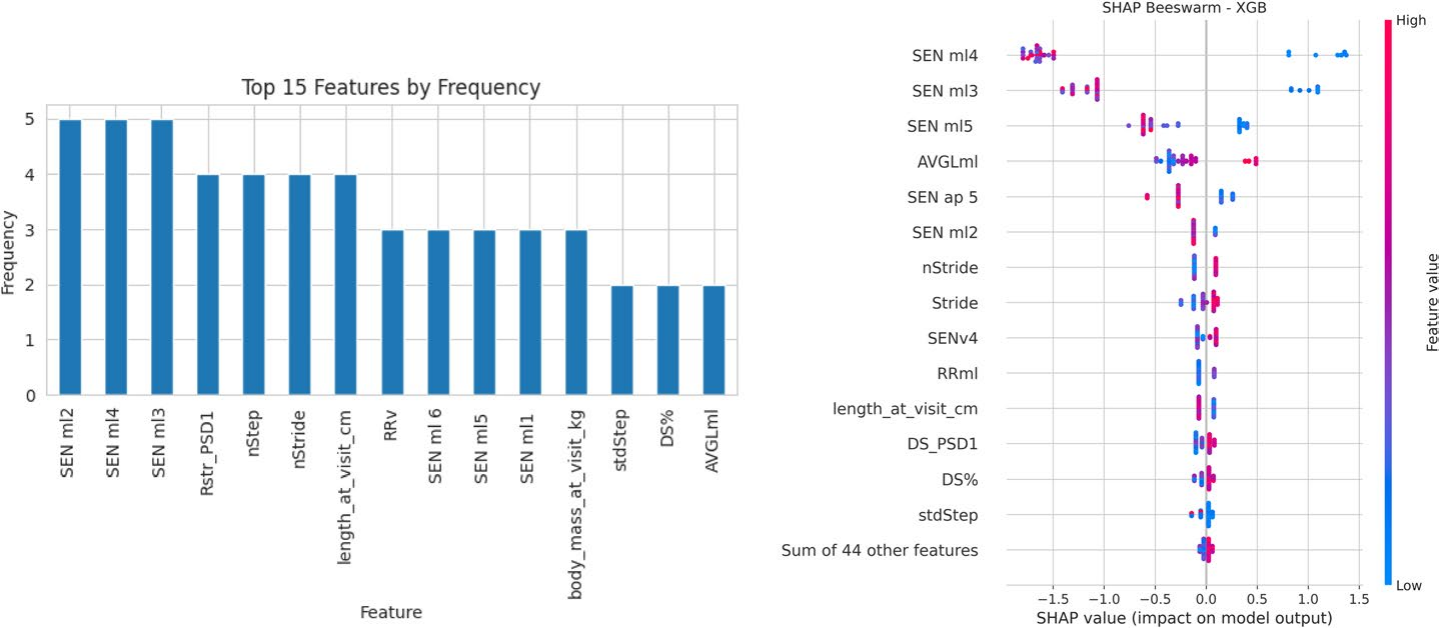
Feature importance for all the models (left), SHAP values for the XGBoost model (right)

Figure 6 presents two visualisations that enhance the interpretability of the model. On the left, it displays the most frequently selected features across the different models, based on the feature importance estimates. On the right, the SHAP values are provided, computed specifically for the above mentioned best-performing model (XGBoost). Children at the highest risk of motor delay are identified by decreased complexity (SEN values) and increased automaticity (AVGl and RR) in the frontal plane (ML and AP directions), suggesting a more automatic and less complex gait pattern than their low-risk peers. In addition, their performance is characterized by lower velocity (i.e., increased nStride and Stride) and stability (i.e., longer DS% phases), combined with higher variability (i.e., increased DS PSD1). They also tend to be smaller (with decreased length and body weight at T24), even when considering corrected age. In general, these results are in agreement with previous work [18], but rather than only describing the differences, they provide further information on the relevance of each parameter and on the set of metrics involved in identifying risk.

## Discussion

In the present work, we proposed a DT framework that integrates heterogenous data collection, quantitative longitudinal motor assessment and advanced AI methods for an efficient holistic data analysis in the context of preterm infants with the final aim of monitoring and understanding their neuromotor trajectory and detect risk of NDDs. First, the main structure of the DT ecosystem is presented, both in terms of model and of functionalities and services provided; then, the framework was applied to a two-points dataset to show how data can be standardised, and as a proof-of-concept to provide an initial demonstration of the feasibility and the advantages of the approach. Results suggest that decreased early motor complexity (lower SEN and higher RQA parameters) is among the most relevant biomarkers of motor delay. These results and their interpretation are supported by findings in the literature on gait [19] and other tasks [16–19, 32–34], reinforcing the vision of the present DT framework, which aims at a longitudinal perspective that overcomes task specificity and instead considers age appropriate tasks within a unified motor trajectory. In addition, reduced motor complexity may limit environmental exploration and potentially impact cognitive development, highlighting the need for the proposed holistic approach to this issue. Although the present results strongly encourage the use of ML, which was able to identify and provide further details with respect to the results of conventional statistics (i.e. relevance of each parameter and set of metrics), we do expect that the use of this approach will provide new insights by identifying non-linear patterns that conventional statistics cannot capture. This advantage becomes especially evident with large, high-dimensional datasets or when relationships are not easily defined or previously known.

### Threats to Validity

The proof-of-concept presented in this paper has some limitations, especially regarding the demonstration of the framework’s longitudinal analytical capabilities. The available data include only two time points (birth and T24) for clinical variables, and a single time point (T24) for motor data. While this is not sufficient to explore meaningful longitudinal changes, the aim here was to show the feasibility of the approach (from the use of wearable sensors in a clinical context to the collection, processing, standardisation, and preliminary integration of multimodal data into the unique reference framework of DT ecosystems). The current proof-of-concept study is therefore intended as a technical and methodological validation rather than a full demonstration of longitudinal analysis. Since we already defined the complete DT model, as more follow-up data become available, the framework will allow for a more in-depth exploration of developmental trajectories and the identification of early biomarkers of NDDs.

Moreover, important contextual factors such as socioeconomic status or parental education levels, which are known to influence child development [35], are not included in the proof-of-concept presented in this paper. This represents a limitation of the present work and of the current data collection. However, the proposed framework is designed to be scalable and adaptable, and future iterations will aim to incorporate additional data sources to capture these relevant environmental and social variables.

### Future Work

This is just a first step towards the ultimate goal of the proposed DT framework, which will enable several types of analysis, e.g., studying how motor complexity evolves in typical and atypical development across different tasks, and identifying if and when it can be used as a predictor of NDDs. Given the relevance of the problem and the absence of uniformed and structured follow-ups of preterm children (motor) development [36], the proposed solution has a potential significant impact in the clinical context.

From a basic science point of view, when fed with longitudinal data as described in the work, the DT will enable the identification of quantitative and interpretative biomarkers of NDDs, advancing our understanding of the conditions and factors that contribute to motor impairments and/or long-term consequences in this population. The DT framework is designed not only to support the discovery of potential biomarkers or causal pathways, but also to be aligned with clinical reasoning and interpretable in real-world settings. For this reason, expert clinical collaboration will continue to guide not only the interpretation of results, but also the formulation of research questions, model definition (e.g. definition of the KG), and validation of analytical outputs, to ensure clinical relevance and usability.

The proposed tool has the potential to support practice by providing clinicians with interpretable, quantitative indicators of motor development and a holistic view of individual cases over time. Once the most sensitive biomarkers to atypical development (e.g., increased risk of NDDs) have been identified and validated, their trajectories could be monitored similarly to how growth charts are used for height and weight. This would allow deviations from typical development to be detected early and interpreted in the context of other relevant variables. Depending on the nature and extent of these deviations, clinicians could be guided toward the most appropriate further assessments or interventions. For example, identifying atypical trunk control or delayed emergence of motor automatisms could help clinicians define therapy plans [37] or specific diagnostic investigations [38]. Moreover, in the future, the proposed framework can be adapted to address additional clinical questions, thanks to the inherent scalability of the DT approach, and integrated with other data sources and processing methods, including: *(i)* new motor performance metrics, both technology-based and based on clinical examinations when available (e.g., standard clinical scales); *(ii)* other infant-related domains that affect their development (e.g., environmental factors such as socio-economic status or parents’ level of education) [35], interventions, diagnoses); *(iii)* alternative data analysis techniques; and *(iv)* extended longitudinal data collection (e.g., from birth to 36 months CA or beyond), including additional timepoints capturing developmental status over time, to allow for a more detailed analysis of individual developmental trajectories.

It can also be tailored for use in centres that collect additional data or only a subset of these data, and adapted for large-cohort, multi-centre studies targeting different infant clinical populations where motor function monitoring is relevant (i.e. from children with rare neurological disorders to all newborns).

## Electronic Supplementary Material

Below is the link to the electronic supplementary material.

Supplementary Material 1

## Data Availability

The data that support the findings of this study are available on request from M.C.B. The data are not publicly available due to privacy/ethical restrictions.

## Footnotes

1 https://fhir.org

2 https://blog.sin-neonatologia.it/wp-content/uploads/2023/04/Manuale-follow-up_sin22_06.04.2023.pdf
